# Performance Evaluation of Malaria Microscopists at Defense Health Facilities in Addis Ababa and Its Surrounding Areas, Ethiopia

**DOI:** 10.1371/journal.pone.0166170

**Published:** 2016-11-28

**Authors:** Tigist Yitbarek, Desalegn Nega, Geremew Tasew, Bineyam Taye, Kassu Desta

**Affiliations:** 1 Emanuel Mental Specialized Hospital, Medical Laboratory Service, Addis Ababa, Ethiopia; 2 Malaria, Other Parasite and Vector Borne Parasitic Diseases Research Team; Ethiopian Public Health Institute, Addis Ababa, Ethiopia; 3 Colgate University, Department of Biology, Hamilton, New York, United States of America; 4 Department of Medical Laboratory Sciences, Collage of Health Sciences, Addis Ababa University, Addis Ababa, Ethiopia; Quensland University of Technology, AUSTRALIA

## Abstract

**Background:**

Blood film microscopy is the gold standard approach for malaria diagnosis, and preferred method for routine patient diagnosis in health facilities. However, the inability of laboratory professionals to correctly detect and identify malaria parasites microscopically leads to an inappropriate administration of anti-malarial drugs to the patients and incorrect findings in research areas. This study was carried out to evaluate the performance of laboratory professionals in malaria diagnosis in health facilities under the Defense Health Main Department in Addis Ababa and its surroundings, Ethiopia.

**Method:**

A cross sectional study was conducted from June to July 2015. Totally, 60 laboratory professionals out of the selected 16 health facilities were included in the study. Data were collected by distributing standardized pre-validated malaria slide-panels and self-administered questionnaires among professionals, onsite in each study facility. Sensitivity, specificity, and strength of agreement (with kappa score) in performance among the study participants against WHO-certified expert malaria microscopists were calculated.

**Result:**

Of the 60 study participants, 8.3% (5/60) correctly read all the distributed slides in terms of parasite detection, species identification and parasite counting; whereas, each of the remaining 55(91.7%) interpreted at least two slides incorrectly. The overall sensitivity and specificity of participants’ performance in detection of malaria parasites were 65.7% and 100%, respectively. Overall, fair agreement (71.4%; Kappa: 0.4) in detection of malaria parasite was observed between the study subjects and expert readers. The overall sensitivity and specificity of participants in species identification of malaria parasites were respectively 41.3% and 100%. Overall, slight agreement (51.1%; kappa: 0.04) in identification of malaria species was observed. Generally, agreement was lower in parasite detection and species identification at low parasite density and mixed infection cases.

**Conclusion:**

The general agreement between the study participants and expert microscopists in malaria parasite detection and species identification was very low, particularly in the cases of low-parasite density and mixed infections. Therefore, regular external quality assessments and further refreshment trainings are crucial to enhance the skill of professionals in malaria microscopy; particularly for those in non-malarious areas where exposure to malaria diagnosis is low.

## Background

Early diagnosis and prompt treatment are key strategies for the management of malaria to reduce mortality and morbidity [1]. There are different diagnostic methods for malaria case management; the common ones are patient‘s clinical assessment, microscopic examination of blood films and the use of malaria rapid tests (RDT). Detection of *Plasmodium* parasites by light microscopy is yet the primary method of malaria diagnosis in most health care facilities throughout the world [2,3]. It has long been the method of choice for diagnosis of many parasitic diseases; particularly, it is considered as the gold standard tool for malaria diagnosis still now. However, microscopy requires good technical skills, quality equipments, reagents and a electric power supply [4,5]. Thus, the quality of this diagnostic method can easily be affected by one of the above determinants.

Correct anti-malarial therapy is critical to delay emergence of drug resistance, and to avoid wastage of other drugs by incorrect prescription, related to incorrect diagnostic result. Confirmatory diagnosis before treatment initiation recently regained attention, highly influenced by the emerging drug resistance and to save more expensive and not easily affordable drugs in the resource-poor countries [4,5]. False positive and false negative malaria diagnostic results produce many consequences up to the death of the patient. In addition, misdiagnosis of malaria results in prescription of inappropriate and high cost drugs for unknown disease, which in turn leads to drug resistance development [6,7].

Low sensitivity and specificity in performance of professionals, in microscopic diagnosis of malaria is a great challenge which has long been described as leading to delayed treatment, development of serious complications, death of patients or exposure to unnecessary treatment [8]. In practice, poor microscopy is a function of multiple factors, including insufficient regular refreshment training for professionals, incorrect blood film preparation techniques, high workload, low quality microscope, and low quality laboratory supplies [9].

Lack of qualified professionals in malaria diagnosis and the lack of regular quality control approaches in the laboratory diagnostic process have been identified as part of possible reasons for low success in malaria control [10]. Many patients are being managed as malaria cases in spite of negative blood film result, which is due to over suspiciousness of health workers or the perceived poor quality of laboratory finding [11].

Compared to malaria endemic areas, the challenge in the quality of malaria microscopy is higher in the health facilities located in the non-malarious areas where laboratory professionals have very low exposure in diagnosing malaria cases. Hence, this study was conducted to assess the capacity of professionals in detecting & identifying malaria parasites on blood film microscopy in the malaria non-endemic Addis Ababa and its surrounding areas, Ethiopia.

## Materials and Methods

### Study area and period

The study was conducted from June to August 2015 in selected health facilities under the Defense Health Main Department (DHMD) of Addis Ababa and its surrounding areas, Ethiopia. Addis Ababa is the capital city of Ethiopia, which is situated in the foothills of the Entoto Mountains and located at an altitude of 2,400 m above sea level. The area is non-malarious highland fringe; however, as the capital city, it serves as a wide trade center where many migrants are coming to-and-fro as a general. The city is an important administrative center not only for the Federal Government of Ethiopia, but also it is a seat for the African Union, International Organizations and United Nations offices for the whole Africa. Therefore, cases of imported malaria are high in cities of this kind and are usually diagnosed incorrectly for parasite detection and species identification due to the low experience of malaria microscopists. Under the DHMD, there are level-based hospitals such as level-1 hospitals, level-2 hospitals, level-3 hospitals, and the armed force referral teaching hospital. Totally 16 health facilities and on average 4 laboratory professionals per each facility were employed to this study.

### Study design and inclusion of study subjects

A cross sectional study design was used by distributing uniformly prepared Giemsa-stained and unstained malaria blood films from different malaria positive donors. Study subjects were all malaria microscopists in the study health facilities, who were available at the diagnostic facilities during data collection. Professionals who were on leave due to sickness and maternal cases, and non-consenting ones were excluded. Total sample size of 60 malaria microscopists participated on this study.

### Data collection process

#### Preparation and distribution of malaria slide panels

Based on the patients consent on the study purpose, 5 milliliters of whole blood was collected from each febrile malaria positive patients who attended the national malaria control center in Adama, Ethiopia; which is 99 km from Addis Ababa. Blood was also collected from malaria negative personnel that had no sign and symptom of malaria and who had no travel history to malaria endemic area. The blood samples were transferred into separate EDTA containing glass tubes.

From the collected blood samples, multiple set of high quality blood film slide panels were prepared; including common malaria parasites (*P*. *falciparum* and *P*. *vivax)*, various parasite densities (low, high), mixed infections and negative blood film slides. Each parasite species of low and high density were collected from unique donors. The Parasite densities were reserved for the study based on their direct count from the donors’ blood; no laboratory dilution was used to make parasite density lower.

Two WHO-prequalified and certified "level-one" expert microscopists in malaria control center were involved in the preparation and validation of malaria slide panels. Both thick (with 6μl of blood) and thin (with 2μl of blood) films were prepared on a single slide. Each slide with thick and thin film was dried overnight, and the thin film was fixed by dipping in absolute methanol. Then, both thin and thick films were stained with 3% Giemsa working solution for 30–45 minutes and arranged in sets. The two experts interpreted the films with three diagnostic keys: (1) the presence/absence of malaria parasite, (2) identification of parasite species and (3) determining parasite density/μl from thick film by counting asexual parasites per 200 leukocytes (or per 500 leukocytes when parasites count was <10), by assuming a standard WBC count of 8,000 leukocytes/μl.

$$Parasite/\mu l=\frac{Number\;of\;asexual\;parasites\;\;\times \;8000\;leukocytes}{200\;leukocytes}$$

#### Administration of blood films

Totally 12 malaria blood film slide panels, 6 Giemsa-stained and 6 unstained slides, were administered for assessment of performance in parasites detection, species identification, and quantification. The standardized malaria blood film slide panels were distributed in the following approach: Two negative stained and unstained slides; 2 *P*. *falciparum* stained and unstained slides of low density (140/μl); 2 *P*. *falciparum* stained and unstained slides of high density (79,640/μl); 2 *P*. *vivax* stained and unstained slides of low density (440/μl); 2 *P*. *vivax* stained and unstained slides of high density (54,400/μl); 2 mixed species (*Pf* + *Pv*) of stained and unstained blood film slides. Blood films were stained with similar Giemsa solution (prepared from similar stock solution and buffer from the central laboratory) by each malaria microscopist under evaluation in all facilities. All study participants used Olympus binocular light microscope equipped with eyepieces (X10) and objectives (x10, x40 and x100), and the same brand immersion oil was used during microscopic examination. Based on WHO recommendation, quantification result of participant was considered correct when it was in between 25% ± the mean calculated from result of expert readers. A total of 120 minutes (10 minutes per each slide) was allocated to examine the 12 malaria slides [4].

#### Data collection tool

Structured questionnaires were used to collect information on the participating facilities and socio-demography of professionals including age, sex, educational status, diagnostic experience, and refreshment trainings. All study participants were formally trained College/University graduated medical laboratory technicians or technologists, and already certified to do malaria microscopy. However, the participants were interviewed for how often they got short-term refreshment trainings aimed to strengthen their skills during their work experience. Such trainings are usually organized by the Ministry of Health (MOH) and/or other nongovernmental organizations (NGO) who are working to support the country health program.

### Data analysis

Data was entered into Microsoft excel sheets and exported to and analyzed using SPSS version 20 for windows. Level of performance in parasite detection, species identification, and quantification was compared with independent demographic parameters. Sensitivity, specificity, and kappa score (to see the strength of an agreement) were calculated to assess performance of laboratory professionals. Based on WHO recommendation, with reference to the percentage agreement in readings between the participants and expert microscopists; malaria microscopists were classified into four:“In-training” (<70% agreement), “Advanced” (≥70%—<80%), “Reference” (≥ 80%—<90%), and “Expert” (≥ 90%). Kappa value was calculated to see strength of agreement. Based on calculation, the strength was classified as: Kappa of <0.20 is slight agreement, 0.21–0.40 is fair agreements, 0.41–0.60 is moderate agreement, 0.61–0.80 is substantial agreement, 0.81–0.99 is almost perfect agreement. Proportional agreement” [(true positive + true negative)/total)] gives a single number to indicate screening performance. This may be the weighted average of the sensitivity and specificity or the weighted average of the positive predictive value and negative predictive value [12,13]. Fisher's exact test was used to assess an association between groups and outcomes, for values smaller than 10 in any cell of the data table.

### Ethical consideration

The study was ethically reviewed and approved by the ethical clearance committee of Addis Ababa University. Informed consent forms were signed by the participating health professionals and blood donors. To ensure confidentiality, the participants’ data was linked to a code number only.

## Result

### Baseline characteristics

Total of 60 laboratory professionals, who were available in the facility during data collection, participated in the study. The mean age of participants was 32 ± 5.43 years and 48(80%) of participants were males. Regarding educational status, 32(53.3%) were bachelor degree and 28(46.7%) were diploma holders. Majority of the study participants that is 38(63.3%) had an experience on malaria microscopy for more than two years and 22(36.7%) had less than two years. Of the total participants, 20(33.3%) took refreshment training on malaria microscopy at least once. Those individuals who participated in an external quality assurance (EQA) program accounted for 36(60%) (Table 1).

**Table 1:** Demography of laboratory professionals under DHMD in Addis Ababa and its surrounding areas, Ethiopia, 2015.

Characteristics	Category	Total study participants (n = 60), %(n)
**Age in year**		
	20–30	40.0(24)
	31–40	46.7(28)
	>40	13.3(8)
**Gender**		
	Male	80(48)
	Female	20(12)
**Educational Status**		
	Bachelor degree	53.3(32)
	Diploma	46.7(28)
**Lab participating in EQA**		
	Yes	60.0(36)
	No	40.0(24)
**In-service refresher training on malaria microscopy**		
	Yes	33.3(20)
	No	66.7(40)
**Frequency of refresher training**		
	Once	33.3% (20)
	No	66.7%(40)
**Experience in routine malaria diagnosis**		
	<2 years	36.7(22)
	≥2 years	63.3(38)

### Performance of laboratory personnel in malaria diagnosis

Of the 60 study participants, only 5(8.3%) interpreted all the distributed slides correctly in terms of parasite detection, species identification and parasite counting; whereas, 55(91.7%), each of which, interpreted at least two slides incorrectly. Based on WHO grading system, 40(66.7%) of participants were at “In-training”, five (8.3%) at “Advanced”, 10 (16.7%) at “Reference”, and five (8.3%) at an “Expert level” in malaria parasite detection (Table 2). From the total, 51(85%) of all participants used not standard quantification system that they reported parasite count in 1+, 2+, 3+ screening system of quantification. The remaining nine (15%) counted parasites against 200 or 500 WBCs and determined the total parasites per the standard 8000 WBCs.

**Table 2:** Agreement between malaria microscopists and experts, Addis Ababa, and its surroundings, 2015.

Parasite Detection		Accuracy In Species Identification		Quantification within 25% of true count	
Grade	n (%)	Grade	n (%)	Grade	n (%)
Expert (≥ 90%)	5(8.3)	Expert (≥ 90%)	5(8.3)	Expert (≥ 50%)	4(6.7)
Reference (≥ 80%)	10(16.7)	Reference (≥ 80%)	0	Reference (≥40%)	8(13.3)
Advanced (≥ 70%)	5(8.3)	Advanced (≥ 70%)	6(10)	Advanced (≥ 30%)	12(20.0)
In-Training (<70%)	40(66.7)	In-Training (<70%)	49(81.7)	In-Training (<30%)	36(60.0)
Total	60(100)	Total	60(100)	Total	60(100)

Abbreviations: n: number; %: percent

**Fig 1:**
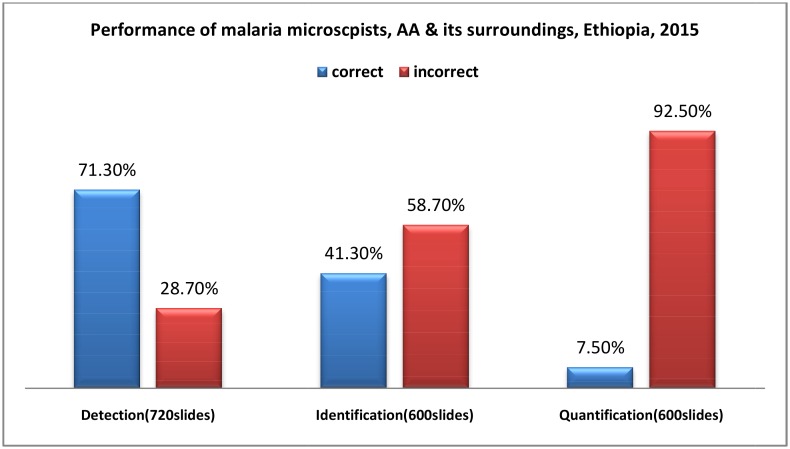
Performance of malaria micrscopists, Addis Ababa, and its surroundings, Ethiopia 2015.

A total of 720 blood film slides of which 240 *P*.*falciparum*, 240 *P*.*vivax*, 120 mixed *Pf + PV*, and 120 negative controls were used for evaluation. From the total 720 slides, 514 (71.3%) were correctly reported for the detection of malaria parasites. Of the 600 positive slides (60 participants x 10 slides), 248 (41.3%) on species identification and only 45 slides (7.5%) on quantification were reported correctly. In the case of negative slides, all of the 120 (60 x 2) slides were reported acceptable (Fig 1)

### Sensitivity and specificity in performance of microscopists

#### Overall reliability

Overall, the sensitivity and specificity of participants’ performance in detection of malaria parasites were 65.7% and 100%, respectively. The overall agreement in detection of malaria parasite between the study subjects and expert reader was 71.4% (Kappa: 0.4) which is 'fair agreement' (Table 3). The overall agreement on identification of malaria species was 51.1% (kappa: 0.04) which is 'slight agreement' (Table 3).

**Table 3:** Over all sensitivity, specificity and agreement in performance of participants in parasite detection and species identification, Ethiopia, 2015.

Participant Reader		Expert Reader		Sensitivity	Specificity	Agreement	Kappa
		Pos	Neg				
**Parasite Detection**	Pos	394	0	65.7%	100%	71.4%	0.4
	Neg	206	120				
	Total	600	120				
**Species Identification**	Correct	248	0	NA	NA	51.1	0.04
	Incorrect	352	120				
	Total	600	120				

Abbreviation: **NA**: Not applicable; **Pos**: positive; **Neg**: Negative

**Fig 2:**
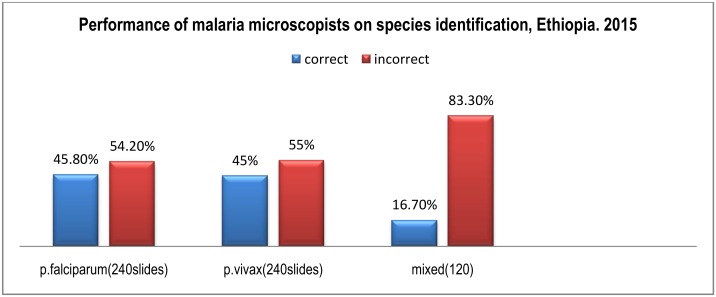
Performance of malaria microscopists in species identification, Ethiopia. 2015.

The performance of participants on species identification showed that 45.8% of blood film slides with *P*. *falciparum*, 45% of slides with *P*.*vivax*, and 16.6% of slides with mixed infections were identified correctly. A total of 100 (83.3%) of slides with mixed infection were identified wrongly as *P*. *falciparum*, 97 (40.4%) of slides with *P*. *falciparum* were identified wrongly as negative and 109 (45.4%) of slides with *P*. *vivax* were identified wrongly as negative (Fig 2).

Of the total 60 participants, the number of participants who scored <80% performance was higher among participants who had not taken refreshment training on malaria microscopy (Table 4).

**Table 4:** Demography related to performance score in malaria species identification, Ethiopia, 2015.

Variables	Category	≥80%*	<80%**	Fisher exact test Two-tailed P-value
Educational Status				
	College Diploma	2	26	1.000
	University Degree	3	29	
Work Experience				
	<2 Years	3	19	0.6998
	≥2 Years	4	34	
Malaria microscopy and QA training				
	Yes	3	17	0.3216
	No	2	38	

Note:

* stands for ‘Passed’;

** stands for ‘Failed’;

QA: Quality Assurance. Fisher's exact test yielded two-tailed P-value is >5%. The association between rows (groups) and columns (outcomes) is considered to be not statistically significant.

#### Performance in relation to parasite density and staining of slides prior to the study

Performance in species identification was 75% and 70% for high-density *P*. *falciparum* stained and unstained slides, respectively. In the case of low-density *P*. *falciparum* slides, species identification was 25% for stained and 13.3% for unstained slides. Overall, the sensitivity and specificity of participants in detection of parasites in the low-density unstained slides were 17.5% and 100%, respectively. The overall agreement was 58.7% (Kappa = 0.17), which is almost ‘slight agreement’ (Table 5).

**Table 5:** Overall performance of participants in relation to slide staining and parasite density, Addis Ababa, Ethiopia, 2015.

Participant Reader		Expert Reader			Sensitivity	Specificity	Agreement	Kappa
		Expected Correct species	Negative	Total				
Stained high density slide	Reported species	120	0	120	100%	100%	100%	1
	Negative	0	120	120				
	Total	120	120	240				
Stained low density slide	Reported species	32	0	32	26.7%	100%	63.3%	0.26
	Negative	88	120	208				
	Total	120	120	240				
Unstained high density slide	Reported species	91	0	91	75.8%	100%	88%	0.76
	Negative	29	120	149				
	Total	120	120	240				
Unstained low density slide	Reported species	21	0	21	17.5%	100%	58.7%	0.17
	Negative	99	120	219				
	Total	120	120	240				

Half of the 720 study blood smears were already stained in the referral laboratory by the expert microscopists and half were unstained before the start of study. Unstained smears were made to be stained by study participants in their laboratories. The participants’ performance in both detection and species identification on pre-study stained slides was better than on the blood smears stained by study participants in their own laboratories.

## Discussion

The present study showed poor performance of professionals in malaria microscopy among diagnostic laboratories at defense health facilities in Addis Ababa and its surrounding areas, Ethiopia. Of the 60 study participants, only 5(8.3%) interpreted all the distributed slides correctly in terms of parasite detection & species identification; and the rest 55 (91.6%), each of which, interpreted at least two slides incorrectly. This is in accordance to study finding from Hawassa, Ethiopia [14].

The sensitivity and specificity of the professionals in detecting malaria parasites were 65.7% and 100%, respectively. The current study showed lower performance in parasite detection when compared to a respective sensitivity and specificity reports of 82% and 96% from Hawassa, Ethiopia [14], 88% and 91% from Zambia [15], 92% and 87% from Uganda [16], and 92% and 90% from USA [17]. The lower sensitivity in detection of parasites indicates that there were high false negative results, which implies high misdiagnosis of true infections. The reason for the high specificity (low false positive rate) is presumably related to the small number of true negative samples in the study. As observed in the current study, 97(40.4%) of *P*. *falciparum* slides, and 109(45.4%) slides with *P*. *vivax* were identified wrongly as negative. The number of participants who failed to correctly report *P*. *falciparum* in the current finding was higher than the 27% failure rate in identification of *P*. *falciparum* in Canada [11] and in consensus with 39% failure rate in species identification in a pooled analysis of the 174 slides in USA [18].

The general performance of participants in species identification is lower than in parasite detection in the current study. This matches the findings from other studies [14,18,19]. From the slides with mixed *P*. *facliparum* and *P*. *vivax* infections, 100 (83.3%) slides were identified wrongly as *P*. *falciparium* mono-infections. This finding is similar to low performance in Hawassa Ethiopia [14], Hong kong [19] and Peruvian Amazon [20]. The possible reasons for this low performance in the current study might be firstly due to the case that study participants were not taking sufficient refreshment training on malaria diagnosis, similar to that reported somewhere else in Ethiopia [21]. Secondly, the study area is less malaria endemic highland area; thus, the professionals had low experience in malaria diagnosis.

In the current study, the overall agreement in detection of malaria parasite between the study subjects and expert readers was 71.4% (Kappa: 0.4), which is categorized as 'fair agreement'. The overall agreement in identification of malaria parasites was 51.1% (kappa: 0.04), which is categorized as 'slight agreement' based on WHO guideline [12,13]. Overall agreement in parasite detection and identification in the current study was lower than the report from malaria endemic areas in Hawassa [14] and Gondar[11], Ethiopia.

In present study, 40(66.7%) of participants were classified as 'In-training' and 5(8.3%) were at expert level; which was lower performance than findings from Hawassa where 17(23.6%) participants were ‘in-training” and the 18(25%) were at expert levels [14], and the finding in Indonesia where nearly all microscopists (95.6%) were at basic or In-training levels, 10(2.3%) were advanced and 9(2.1%) were reference microscopists [22]. This difference may be due to the difference in malaria epidemiology in the stated areas.

There were a big difference in parasite detection and species identification in terms of parasite density, and staining of blood film. Performance in species identification was better on high density stained blood film slides (75%) than on the low density (25%) stained *P*. *falciparum* slides. Performance in species identification was better on high-density unstained (70%) than on the low density (13.3%) unstained *P*. *falciparum* slides. The study clearly showed the microscopists had low experience in staining of malaria slide smears and had low capacity in malaria diagnosis, particularly in the case of low parasite density. The overall agreement in detection of malaria parasites on low density stained slides was 63.3% (Kappa: 0.26) which is lower than an agreement of 100% in high density stained slide. The overall agreement on low density unstained slides was 58.7% (Kappa: 0.17) and an agreement on unstained slide with high density was 88% (kappa: 0.76).

The current study brought the first evidence on performance of malaria microscopists in Addis Ababa and its surrounding areas. However, it has come with some limitations, as it could not include all malaria microscopists in the study sites; only those who were available during the time of data collection were recruited. The species of malaria parasites used for malaria blood film slide panel preparation were not characterized or molecularly typed by polymerase chain reaction, only identified by certified laboratory technologists.

## Conclusion

The present study showed poor performance of the study participants in malaria microscopy among the diagnostic laboratories at defense health facilities in Addis Ababa and its surrounding areas, Ethiopia. The general agreement between the study participants and expert microscopists in malaria parasite detection and species identification was very low, particularly in the cases of low parasite density and mixed infections. The misinterpretation in malaria diagnosis in turn equally results in misadministration of inappropriate treatment to the patients. This may direct to emergence of drug resistance, exposing patients to suffer from repeated diseases and death, economical loss and wastage of costly drugs. Therefore, to handle these challenges, further regular external quality assessments and trainings are crucial for career competency of professionals, particularly to those who are working in malaria non-endemic areas.

## Supporting Information

S1 File Study Questionnaire. (DOCX)

## Abbreviations

SPSS: Statistical package for social science
RDT: rapid diagnostic tests
DHMD: Defense Health Main Department
WHO: World health organization
WBCs: White blood cells
Pf: *P*.*falciparum*
Pv: *P*.*vivax*
EQA: external quality assurance
AA: Addis Ababa
