# Supplementary material for: Performance Evaluation of Malaria Microscopists at Defense Health Facilities in Addis Ababa and Its Surrounding Areas, Ethiopia
Source: PLoS One. 2016 Nov 28;11(11):e0166170. doi: 10.1371/journal.pone.0166170 (PMC5125591; doi:10.1371/journal.pone.0166170)
Supplement: S1 File — (DOCX) [file pone.0166170.s001.docx]

# Annex I: Questionnaire

| **serial** | **Question** | **Response Category** | **Remark** |
| --- | --- | --- | --- |
| 1 | Age in Years | _______________ |  |
| 2 | Sex | 1. Male 2. Female |  |
| 3 | When did you Graduate? | 1. Less than 1yr 2. 1-2 years 3. More than 2 years______ |  |
| 4 | Where did you graduate? | 1. Government 2. Private 3. Other_______ |  |
| 5 | What did you graduate with? | 1. Diploma  2.BSC. |  |
| 6 | Is the lab provide routine malaria smear microscopy service | 1. yes  2. no |  |
| 7 | How long has it been since you started working on malaria microscopy? | 1. <2 years 2. ≥2 years |  |
| 8 | How many malaria slides do you examine daily? | 1. Less than 5 2. From 5-10 3. More than 10___ |  |
| 9 | Have you ever had an in-service training on malaria microscopy? | 1. Yes 2. No |  |
| 10 | If yes, for how long? | ___________________ |  |
| 11 | If yes, who gave you the training? | 1. Government? 2. NGO |  |
| 12 | Is there any supervision from regional or national laboratories | 1. Yes 2. No |  |
| 13 | Which of microscope or RDT do u prefer | _______________ |  |
| 14 | Does your lab. participate in EQA programs? | 1.yes  2. no |  |
| 15 | Is the staining solution are accessible and stored in appropriate place | 1. Yes 2. No |  |
| 16 | Is the microscope functional properly? | 1. Yes 2. No |  |
| 17 | What method do you use for the diagnosis of malaria | 1. Thin film only 2. Thick film only 3. Thin and thick film |  |
| 18 | Do you perform parasitemia? | 1.yes  2.no |  |
| 19 | If yes, which methods do you use | 1. +, ++, +++ 2. Parasite per micro liter/ WBC 3. Parasite per micro liter/ RBC |  |

# Annex III: Lab Result reporting format

**Result reporting form for laboratory professionals**

Lab technician Lab technologist

| **Slide ID** | **Result** | | | | **Remark** |
| --- | --- | --- | --- | --- | --- |
|  | **Negative** | **Positive** | | |  |
|  |  | **Species** | **Stage** | **Parasite Load** |  |
|  |  |  |  |  |  |
|  |  |  |  |  |  |
|  |  |  |  |  |  |
|  |  |  |  |  |  |
|  |  |  |  |  |  |
|  |  |  |  |  |  |
|  |  |  |  |  |  |
|  |  |  |  |  |  |
|  |  |  |  |  |  |
|  |  |  |  |  |  |
|  |  |  |  |  |  |
|  |  |  |  |  |  |

Date: ________________

Signature of the lab technicians/technologist _________________________
